# The Role of *Moringa oleifera* in Caries Prevention and Enamel Remineralization

**DOI:** 10.1055/s-0045-1814465

**Published:** 2026-01-14

**Authors:** Hanadi S. Lingawi

**Affiliations:** 1 Department of Preventive Dentistry, Faculty of Dental Medicine, Umm Al-Qura University, Makkah, Saudi Arabia

**Keywords:** *Moringa oleifera*, dental caries, enamel, remineralization

## Abstract

*Moringa oleifera (M. oleifera)* is a plant of significant medical interest because of its abundance of bioactive compounds that exhibit antibacterial, anti-inflammatory, and antioxidant properties. Leaf extracts of *M. oleifera* are also rich in essential minerals and proteins, highlighting their potential as natural therapeutic agents for use in oral health care. Recent investigations have explored the efficacy of these extracts in the prevention of dental caries and the promotion of dental enamel remineralization. This review aimed to consolidate the current evidence regarding the role of *M. oleifera* in caries prevention and enamel remineralization potential, to provide a critical evaluation of the strengths and limitations of the current literature while offering insights into the plant's therapeutic prospects as a natural agent for use in caries prevention and enamel remineralization. The review was achieved through an extensive literature search conducted across the PubMed, Science Direct, Scopus, and Google Scholar databases. The search was limited to original research articles, systematic reviews, and peer-reviewed publications written in English from 2005 to 2025. Letters to the editor, editorials, and case reports were excluded. While the preliminary findings indicated a promising therapeutic potential for *M. oleifera* in terms of preventing dental caries and promoting enamel remineralization, the existing literature is constrained by several limitations, including a lack of clinical trials, heterogeneity in the methodologies used, short study durations, and insufficient understanding of the underlying mechanisms. In conclusion, robust in vivo studies and collaborative efforts between researchers and clinicians are essential to facilitate the translation of laboratory findings regarding *M. oleifera* extracts into clinical dentistry applications.

## Introduction

Dental caries development is a complicated, multifactorial process that leads to demineralization of dental hard tissues caused by acid produced by bacteria. The development of dental caries requires the presence of cariogenic bacteria, dietary carbohydrates, vulnerable tooth surfaces, and adequate time. 1 If left untreated, dental caries can progress to affect wider and deeper tooth structures, causing pain, infection, and potential tooth loss.

Caries arises because of the activity of several oral bacterial species. Among these, *Streptococcus mutans (S. mutans)* is considered one of the most cariogenic bacteria in the oral environment. Other common cariogenic species include different strains of *Streptococcus* bacteria, *Lactobacillus* spp., and *Actinomyces* spp. In a healthy oral cavity, the oral microbiota maintains a state of symbiosis that is essential for caries prevention and continued oral health. However, this balance can be disrupted by many factors, such as poor oral hygiene, high carbohydrate intake, reduced salivary flow, and low fluoride exposure, which ultimately promote enamel demineralization.

Oral diseases affect approximately 3.5 billion people globally, as documented in the Global Oral Health Status report, published by the World Health Organization (WHO) for the year 2022. Of these individuals, nearly 2 billion suffer from caries in their permanent dentition, and around 514 million children have caries in their primary dentition. 2 3 Dental caries is the most prevalent noninfectious disease in children. 3 As such, it is considered a global public health issue that requires the establishment of comprehensive and evidence-based preventive strategies. 4 Therefore, community-based methods, such as water and milk fluoridation, the adoption of fluoridated toothpastes and mouthwashes, and the use of fissure sealants have been introduced and proven to be effective methods toward caries prevention. 5 6 However, concerns remain regarding the potential risks associated with excessive fluoride exposure, and this has increased the interest in finding fluoridation alternatives. Advanced technologies now focus on regenerative, biomimetic strategies that simulate the natural processes of enamel repair and offer promising approaches for minimally invasive caries management. 7

One approach for managing caries is the use of antimicrobial agents such as chlorhexidine mouthwash. These agents have shown efficacy in reducing *S. mutans* levels; however, their long-term use is associated with adverse effects, including microbial resistance. Consequently, research has increasingly shifted toward the study of natural plant-derived compounds as safer and more sustainable alternatives. 8 Many natural products offer the potential to improve oral health with fewer side effects than chemical agents and have demonstrated efficacy against both dental caries and periodontal diseases. 9

While conventional therapies remain essential, the exploration of plant-derived compounds as alternative oral care agents is important. These compounds are already traditionally used by nearly 80% of the global population in the rural regions of developing countries due to their accessibility, cost-effectiveness, and biocompatibility. 10

One plant that has attracted particular attention for its potential oral health benefits is *Moringa oleifera (M. oleifera)* , a plant rich in bioactive compounds that may aid in preventing dental caries and periodontal diseases. 11 While most of the currently published literature review articles have been concerned with the effects of *M. oleifera* on oral and periodontal health. The aim of this narrative review was to consolidate the existing scientific evidence regarding the role of *M. oleifera* in caries prevention, with particular emphasis on its enamel remineralization potential to provide a critical evaluation of the strengths and limitations of the current literature while offering insights into the plant's therapeutic prospects as a natural agent for use in caries prevention and enamel remineralization.

## Search Strategy

The terms “ *Moringa oleifera* ,” “dental caries,” “remineralization,” and “dental enamel” were used as keywords in an electronic search in the Medline [PubMed interface], Science Direct, Scopus, and Google Scholar databases. The search was limited to original research articles, systematic reviews, and peer-reviewed texts published between January 2005 and June 2025. Materials published as letters to the editors, editorials, and case reports were excluded. Only works published in English were included. The abstracts were evaluated for eligibility, duplicates were removed, and the data were analyzed.

## Background on *Moringa oleifera*

*M. oleifera* is a fast-growing, drought-resistant tree belonging to the Moringaceae family. It is native to tropical and subtropical regions, with particularly widespread growth in northern India, Southeast and East Asia, the Red Sea region, and Madagascar. 12 India is currently recognized as the world's largest producer of *M. oleifera* trees. Common English names for the tree include “drumstick tree” (referring to the shape of its seedpods), “horseradish tree” (due to the pungent taste of its roots), and “miracle tree,” highlighting the nutritional and medicinal value of its various parts.

The plant contains over 92 bioactive compounds distributed throughout its flowers, fruits, leaves, stems, roots, and seeds. 13 *M. oleifera* leaves and seeds are particularly favored as food supplements because of their characteristic flavor 14 15 and because of their extended shelf life, as they undergo only minimal degradation of their nutritional contents during storage. 16 In many countries, where resources are limited, *M. oleifera* serves as a good source of macro- and micronutrients. It is rich in protein, carbohydrates, fiber, essential minerals, and vitamins. The dried leaves are particularly rich in vitamin E, vitamin C, β-carotene, calcium, potassium, magnesium, and iron, making them excellent sources of nutrients and antioxidants. 17 18

The pharmacological properties of *M. oleifera* extracts, including their anti-inflammatory, anti-bacterial, anticancer activities, are well documented. 19 20 They are primarily attributed to their phytochemical components. Research indicates that different parts of *M. oleifera* contain distinct groups of compounds that are unique to each specific part. For example, the stems contain different alkaloid compounds such as moringinine and moringin; the flowers are rich in flavonoids, sucrose, alkaloids, and amino acids, while the fruits are rich in cytokines. 19 20

The pharmacological properties of *M. oleifera* extracts, including their anti-inflammatory, antibacterial, and anticancer activities, are well documented. 19 20 They are primarily attributed to their phytochemical components. Research indicates that different parts of *M. oleifera* contain distinct groups of compounds that are unique to each specific part. For instance, the stems contain different alkaloid compounds such as moringinine and moringin, the flowers are rich in flavonoids, sucrose, alkaloids, and amino acids while the fruits are rich in cytokines. 19 20

In traditional medicine, preparations derived from *M. oleifera* leaves and seeds have been widely used in place of conventional pharmaceutical agents, as they are more affordable, less toxic, and show satisfactory therapeutic efficacy. 21 The leaves, in particular, are traditionally used in the treatment of wounds, rheumatism, arthritis, diarrhea, and malaria. 22 The seeds are usually incorporated into skincare formulations or used in preparations for the treatment of gastrointestinal issues and for the treatment of anemia. 23 The roots are valued for their wound-healing effects and are used in the management of respiratory conditions such as asthma and bronchitis. 23 Beyond these medicinal applications, *M. oleifera* has also been used in other fields, ranging from water purification to agriculture, and in many industrial processes, highlighting its importance as a multifunctional plant. 24

## Role of *Moringa oleifera* in Caries Prevention

The anti-caries properties of *M. oleifera* are primarily mediated through two key mechanisms. The first mechanism involves the inhibition of enamel demineralization, which is typically facilitated by the anti-microbial, antioxidant, and anti-inflammatory characteristics of *M. oleifera* extracts. 25 The second mechanism is related to the promotion of enamel remineralization and hydroxyapatite crystal formation, through increased concentrations of minerals in the oral environment due to the release of calcium and phosphate ions from *M. oleifera* extracts. 7 26

### Antimicrobial Effects

The effect of *M. oleifera* extracts is primarily attributed to their direct inhibitory action against the growth of a wide range of cariogenic bacteria, including *S. mutans* , *S. salivarius* , *Lactobacillus acidophilus* , and *Staphylococcus aureus* . 27 This antimicrobial activity is mediated by a range of bioactive phytochemicals, including saponins, flavonoids, tannins, alkaloids (e.g., terpenoids), and phenolic compounds. 25 Saponins exert antibacterial effects by disturbing bacterial cell membrane permeability, which compromises membrane integrity and causes cell lysis. 28 29 In contrast, flavonoids interfere with bacterial protein function by forming hydrogen-bonded complexes that impair bacterial metabolism and physiological processes. They also inhibit bacterial cell wall synthesis through collective cytotoxic effects. 30 31 In the case of alkaloids, Cushnie et al have proposed that the antibacterial effects arise because of the ability of alkaloids to disrupt the peptidoglycan matrix of the bacterial cell walls, leading to weakening of the wall integrity and bacteriolysis. Conversely, terpenoids impair bacterial growth through their interactions with porins on the bacterial outer membrane, which reduces membrane permeability and restricts nutrient intake. 32

Multiple in vitro studies have demonstrated that *M. oleifera* leaf extracts, particularly ethanol extracts, exhibit a significant inhibitory effect against *S. mutans* . Fouad and Fouad reported that ethanol extracts produced significantly larger inhibition zones than aqueous extracts. 33 Also, a study by Jwa demonstrated that both aqueous and ethanol extracts suppress *S. mutans* growth and biofilm formation, with ethanol extract showing stronger anti-biofilm effects. 34 This difference most likely emerges due to the chemical structure of ethanol, as the hydroxyl groups in ethanol provide better binding with polar compounds like flavonoids, resulting in the dissolution of higher concentrations of flavonoids in ethanol extracts. These increased flavonoid concentrations enhance the antibacterial activity of the *M. oleifera* extracts.

However, the use of ethanol as a solvent may present a compromise when it comes to remineralization properties. Aqueous solutions, being more effective at dissolving mineral salts such as calcium and phosphate ions, are preferred for their ability to deliver these essential ions for enamel remineralization. Ethanol extracts, while richer in flavonoids, may have a reduced concentration of calcium and phosphate ions due to the solvent's lower affinity for these minerals. This reduction in concentration may slightly reduce the remineralizing potential of ethanol extract compared with aqueous extracts. 22 23 25

Nevertheless, the overall clinical efficacy of *M. oleifera* extracts for dental caries prevention and treatment may depend on a balanced formulation that combines both the antibacterial properties of ethanol-based extracts and the remineralizing benefits of aqueous extracts, potentially through co-delivery systems that address both aspects simultaneously.

Elgamily et al tested leaf, seed, and root extract effects on the growth of *S. mutans and* found that *M. oleifera* leaf extracts showed the highest antibacterial activity. 27 This probably reflects the high leaf concentrations of bioactive compounds, such as flavonoids and polyphenols, which have strong antimicrobial properties. Because these compounds are more abundant in the leaves, leaf extracts show enhanced effectiveness against cariogenic bacteria compared with extracts from other plant parts, such as roots and seeds. 20 22 25

### Antioxidant Effects

The presence of large quantities of cariogenic bacteria, such as *S. mutans,* in the oral cavity triggers the body's immune response, leading to the activation of neutrophils and macrophages. As part of the host defense mechanism, these immune cells produce reactive oxygen species (ROS), such as hydrogen peroxide and superoxide. While ROS helps protect the oral environment by killing pathogens, excessive or prolonged ROS production can overwhelm the defense system in the oral cavity and lead to oxidative stress. The excess ROS can then begin to damage the host cells in addition to the pathogens. The enamel demineralization process is particularly affected by oxidative stress, as ROS encourage mineral loss and weaken the enamel, making it more vulnerable to dental caries. *M. oleifera* is rich in polyphenolic compounds, such as quercetin and kaempferol, which are both known for their antioxidant properties. This antioxidative activity aids in reducing oxidative stress and, accordingly, suppressing enamel demineralization, thereby protecting teeth against dental caries formation. 22

### Anti-inflammatory Effects

Inflammation is a key factor in enamel degeneration. The release of proinflammatory cytokines, such as tumor necrosis factor-α (TMF-α) and interleukin-1β (IL-1β), in response to bacterial infection of the oral cavity, stimulates biofilm formation and enhances acidogenic activity by promoting the proliferation of acidogenic caries-inducing microorganisms, including *S. mutans* and *Lactobacillus* species. 35 These cytokines also reduce the salivary buffering capacity, creating an environment that favors enamel demineralization. 35

*M. oleifera* has substantial anti-inflammatory properties, primarily attributed to bioactive compounds, such as sulforaphane and the flavonoid kaempferol. These phytochemicals are implicated in modulating inflammatory pathways within the oral cavity and potentially reducing acidogenesis, thereby promoting an environment conducive to enamel remineralization. 35 Sulforaphane exerts anti-inflammatory effects primarily through inhibition of the NF-kB signaling pathway, thus suppressing the transcription of proinflammatory mediators such as Cox-2 and cytokines, including TNF-α and IL-1β. Kaempferol complements these effects by inhibiting the phosphorylation and nuclear transformation of NF-kB p65 subunits as well as downregulating the MAPK signaling cascade. 35

## Role of *Moringa oleifera* in Dental Enamel Remineralization

Dental enamel is a highly mineralized and acellular dental tissue that consists of ∼98 wt.% of calcium hydroxyapatite [CA _10_ (PO _4_ ) _6_ OH _2_ ] with various impurities and 2% organic matrix and water. 36 The enamel remineralization process involves the restoration of lost mineral content, mainly calcium and phosphate ions, to repair the damage to the enamel structure caused by acids from cariogenic bacteria, food, or other sources. Both ions (calcium and phosphate) must be present in sufficient concentrations in the oral environment for the remineralization process to occur. If the ion levels are insufficient because of poor salivary flow, acidic conditions, or poor dietary intake, then the process of enamel demineralization may continue. 37 38

The content of both calcium and phosphate ions is high in leaf extracts of *M. oleifera* , which can contain ∼400 to 500 mg of calcium and 50 to 100 mg of phosphate per 100 g of dry weight. These high concentrations of calcium and phosphate ions provide a direct source of ions for initiating the remineralization process in dental enamel. 20 The leaf extracts also contain miscellaneous amino acids, flavonoids, polyphenols, and saponins, which may act synergistically to promote the remineralization of dental enamel. The flavonoids and phenolic compounds present in the *M. oleifera* extract impart antioxidant properties and facilitate a peptide-guided mineral nucleation environment that promotes the formation of a scaffold for hydroxyapatite crystal regrowth. 33 Ethanol-based *M. oleifera* extracts have also demonstrated significant antimicrobial activity, particularly against *S. mutans* , as indicated by the inhibition of biofilm formation and reduction in the acid challenge responsible for enamel demineralization. Dental smear and surface layer studies have also shown that the saponins in *M. oleifera* are capable of dissolving outer organic/inorganic debris to enable better mineral penetration and enamel remineralization. 35

Essam Eliwa et al compared the in vitro enamel remineralization of artificially demineralized primary teeth after treatment with an *M. oleifera* hydrogel versus an eggshell or sodium fluoride. Their subsequent scanning electron microscopy (SEM) and energy dispersive X-ray (EXD) examinations confirmed that the *M. oleifera* hydrogel treatment significantly increased the calcium and phosphate deposition beyond that achieved using either the fluoride or eggshell hydrogel treatments, confirming better remineralization provided by the *M. oleifera* hydrogel. 39

Younis et al also used SEM and EXD to evaluate the in vitro effects of treatments with a lyophilized *M. oleifera* leaf extract loaded into a varnish. 7 The *M. oleifera* treatment showed potential for promoting the regeneration of enamel surface lesions, as the mineral constituents of the extract chemically interacted with enamel hydroxyapatite crystals to facilitate remineralization. Furthermore, peptide-mediated pathways activated the capacity to guide the formation of a mineralized layer that was structurally similar to natural enamel. 7

Al-Sadek et al compared the effect of twice daily application of an aqueous *M. oleifera* extract versus casein phosphopeptide amorphous calcium phosphate (CPP-ACP) for 1 week on primary human enamel. 40 SEM and EXD analysis demonstrated a greater increase in calcium and phosphorus deposition in teeth treated with the *M. oleifera* extract than with CPP-ACP, indicating the superior remineralization potential of the plant extract. 40 Khalaf et al compared the remineralization effect of a topical *M. oleifera* extract application to a fluoride paste application in extracted permanent teeth from uremic renal failure patients—a population particularly prone to dental caries. 41 SEM and EXD measurements showed higher enamel calcium and phosphate levels in the specimens treated with the *M. oleifera* extract than with the fluoride paste, suggesting that the *M. oleifera* extract offered better caries protection even in medically compromised patients. 41

Comparisons of the effects of *M. oleifera* extracts on artificially demineralized enamel and dentine, to those of other natural plant extracts, such as green or black tea, have also indicated a superior effect for *M. oleifera* , suggesting that *M. oleifera* extracts have the capability to initiate the remineralization process and can be viewed as effective natural remineralizing agents. 42 However, no human clinical trials have yet been conducted; so, these findings remain limited to those obtained using ex vivo or animal models.

The proposed mechanism by which *M. oleifera* extracts enhance enamel mineralization involves a combination of several complementary actions. First, the *M. oleifera* extract serves as a rich source of calcium and phosphate ions, thereby directly facilitating the deposition of minerals into enamel lesions. At the same time, the amino acids present in the extract may act similarly to an enamel matrix peptide and guide the nucleation process necessary for effective remineralization. The extract also exhibits antimicrobial properties, as the resulting inhibition of *S. mutans* and the suppression of biofilm formation could reduce the local acidity factors that contribute to demineralization. Furthermore, saponins in the extract can assist in surface cleaning by removing debris and smear layers and improving the penetration of the extract into the enamel. Together, these actions function synergistically to restore the enamel mineral content more effectively than is observed using current conventional agents.

## Limitations and Future Directions

*M. oleifera* has gained significant attention in recent dental research because of its potential antimicrobial, antioxidant, and pro-remineralization effects. Several in vitro studies have explored the application of *M. oleifera* extracts as a dental caries prevention method and for the enhancement of enamel remineralization. Although the primary findings appear promising, notable limitations still hinder the translation of these results into clinical practice.

1. *The limited scope and number of studies* : The number of peer-reviewed studies that have specifically evaluated the effect of *M. oleifera* extract on dental caries prevention and enamel remineralization is limited. Most of the available research is exploratory and has often been published in regional journals with limited reach. This creates difficulties in establishing a comprehensive understanding of the efficacy of *M. oleifera* extracts as a dental caries treatment.
2. *The predominance of in vitro study designs* : A major limitation is the reliance on in vitro experimental models, which fail to replicate the complexity of the oral environment, including factors such as saliva composition, dynamic pH changes, oral microbiota, biofilm activity, and dietary habits. All these factors play critical in vivo roles in caries development and remineralization; therefore, findings from laboratory-based studies may not accurately predict the effectiveness of *M* . *oleifera* in in vivo-based interventions.
3. *Methodological inconsistencies* : A lack of standardization exists across studies with respect to the extraction methods (aqueous, methanolic, ethanolic), the plant parts used (leaves, seeds, stems), the extract concentrations and formulations, the duration and frequency of the applications, and the data analysis methods. These methodological variations significantly reduce the ability to compare the findings of the various studies.
4. *Short-term evaluation periods* : Most studies have assessed outcomes over short durations, typically ranging from 24 hours to a few weeks. Thus, the long-term effects, including prolonged remineralization, enamel resistance, and caries recurrence, have not been studied. Without longitudinal data, the durability and consistency of *M* . *oleifera* effects remain unknown.
5. *Unclear mechanisms of action* : While *M* . *oleifera* clearly possesses bioactive components that could theoretically aid in enamel repair and microbial inhibition, the mechanisms underlying these effects are poorly defined. Whether the observed remineralization is due to mineral deposition, protein interaction with enamel, or antimicrobial effects on the dental plaque layer remains unclear, based on the current evidence.
6. *Lack of clinical trials* : To date, minimal or no clinical research has evaluated *M* . *oleifera* extracts for dental use as anticaries agents in human subjects. The absence of clinical trials represents a major research gap, as in vivo or animal findings cannot be directly applied to human populations without robust clinical validation.
7. *Limited safety and toxicological assessments* : Few studies have assessed the biocompatibility and safety of *M* . *oleifera* extracts when used intraorally over prolonged periods. Before the incorporation of *M* . *oleifera* extracts into commercial oral care products, comprehensive toxicological studies are necessary to ensure safety, particularly regarding potential allergic reactions, mucosal irritation, or systemic absorption.

## Conclusions

*M* . *oleifera* is a potent source of bioactive compounds with demonstrated antibacterial, anti-inflammatory, and antioxidant properties. In particular, its leaf extracts, characterized by high mineral and protein contents, show promising potential as natural therapeutic agents for the prevention of dental caries and the promotion of enamel remineralization. However, as this literature review shows, the current evidence is constrained by a scarcity of clinical studies, as well as methodological inconsistencies, short study durations, and insufficient understanding of the underlying mechanisms of action.

To validate the therapeutic efficacy and clinical relevance of *M. oleifera* extracts in dental caries prevention and enamel remineralization, future research should prioritize the development of standardized extract formulations, the performance of long-term in vivo and clinical studies, and the elucidation of the molecular mechanisms of action involved. Furthermore, robust in vivo studies and collaborative efforts between researchers and clinicians are essential to facilitate the translation of laboratory findings into effective clinical applications.
